# Whole blood transcriptome characterization of young female triathlon athletes following an endurance exercise: a pilot study

**DOI:** 10.1152/physiolgenomics.00090.2022

**Published:** 2022-10-17

**Authors:** Attila Bácsi, András Penyige, Gergely Becs, Szilvia Benkő, Elek Gergő Kovács, Csaba Jenei, István Pócsi, József Balla, László Csernoch, Ildikó Balatoni

**Affiliations:** ^1^Department of Immunology, Faculty of Medicine, University of Debrecen, Debrecen, Hungary; ^2^Department of Human Genetics, Faculty of Medicine, Faculty of Pharmacy, University of Debrecen, Debrecen, Hungary; ^3^Division of Nephrology, Department of Internal Medicine, Faculty of Medicine, University of Debrecen, Debrecen, Hungary; ^4^Department of Physiology, Faculty of Medicine, University of Debrecen, Debrecen, Hungary; ^5^Doctoral School of Molecular Cellular and Immune Biology, Faculty of Medicine, University of Debrecen, Debrecen, Hungary; ^6^Department of Cardiology, Faculty of Medicine, University of Debrecen, Debrecen, Hungary; ^7^Department of Molecular Biotechnology and Microbiology, Institute of Biotechnology, Faculty of Science and Technology, University of Debrecen, Debrecen, Hungary; ^8^Department of Internal Medicine, Faculty of Medicine, University of Debrecen, Debrecen, Hungary; ^9^Clinical Center, University of Debrecen, Debrecen, Hungary

**Keywords:** endurance exercise, ImSig, RNA sequencing, whole blood transcriptome, young female athletes

## Abstract

The vast majority of studies focusing on the effects of endurance exercise on hematological parameters and leukocyte gene expression were performed in adult men, so our aim was to investigate these changes in young females. Four young (age 15.3 ± 1.3 yr) elite female athletes completed an exercise session, in which they accomplished the cycling and running disciplines of a junior triathlon race. Blood samples were taken immediately before the exercise, right after the exercise, and then 1, 2, and 7 days later. Analysis of cell counts and routine biochemical parameters were complemented by RNA sequencing (RNA-seq) to whole blood samples. The applied exercise load did not trigger remarkable changes in either cardiovascular or biochemical parameters; however, it caused a significant increase in the percentage of neutrophils and a significant reduction in the ratio of lymphocytes immediately after exercise. Furthermore, endurance exercise induced a characteristic gene expression pattern change in the blood transcriptome. Gene set enrichment analysis (GSEA) using the Reactome database revealed that the expression of genes involved in immune processes and neutrophil granulocyte activation was upregulated, whereas the expression of genes important in translation and rRNA metabolism was downregulated. Comparison of a set of immune cell gene signatures (ImSig) and our transcriptomic data identified 15 overlapping genes related to T-cell functions and involved in podosome formation and adhesion to the vessel wall. Our results suggest that RNA-seq to whole blood together with ImSig analysis are useful tools for the investigation of systemic responses to endurance exercise.

## INTRODUCTION

A large and growing body of evidence demonstrates that regular physical activity provides numerous positive effects on human health and can prevent diseases such as hypertension, type 2 diabetes, coronary heart disease, and cancer (1). However, the mechanisms by which the different types, amounts, and intensities of exercise induce these benefits are not yet fully understood. Investigation of the cellular and molecular responses using comprehensive transcriptome analysis by microarray techniques or RNA sequencing (RNA-seq) methodology represents an important way to improve our knowledge about systemic effects of exercise training. Indeed, several studies have utilized microarrays (2, 3) and RNA-seq (4, 5) to evaluate changes in the transcription profile of human skeletal muscle induced by divergent exercise stimuli. Moreover, in studies focusing on the impact of physical activity on obesity RNA-seq was applied to determine the gene expression profile by which adipose tissue responds to acute and chronic exercise (6, 7).

Systemic responses to physical activity not only involve adaptation of the skeletal muscle and adipose tissue to changes, but also that of peripheral blood. Blood cells dynamically react to the stress stimuli triggered by exercise training, therefore blood samples are often the subjects of gene expression analyses. Depending on the aims of the study, whole blood, white blood cells, peripheral blood mononuclear cells, or isolated subpopulations of leukocytes such as lymphocytes and monocytes were profiled (reviewed in Ref. 8). However, it is important to note that vast majority of the published data sets related to exercise training and blood global mRNA profiling were generated using microarray technology.

In this study, we utilized RNA-seq to whole blood samples of four young female triathlon athletes, to explore transcriptome‐wide changes in gene expression and their kinetics induced by endurance exercise mimicking the cycling and running disciplines of a junior triathlon race.

## MATERIALS AND METHODS

### Subjects

The study was approved by the Medical Research Council (No. 3890-8/2018/EÜIG) and complied with the guidelines set forth in the Declaration of Helsinki. All participants provided written informed consent to participate in the study.

The experiments were carried out with the participation of the female Hungarian Junior Triathlon Team. The four subjects took part in a 4-wk-long (for individual differences due to synchronization see below in this section) session during the off-season training period in the winter of 2019. They followed the same daily routine of 1-h low-intensity training in the morning and in the afternoon to maintain physical fitness. They kept the same diet as recommended by the coaching team. The actual test was carried out on the 5th day following the start of the menstrual bleeding to synchronize the hormonal status of the participants and to avoid any possible interference of the ovulation during the restitution period.

### Exercise Session

The exercise session was meant to mimic the cycling and running disciplines of a junior triathlon race (20 km bike and 5 km run https://www.triathlon.org/). Thus, the participants cycled for 30 min using their own bicycles (on a special stand) and then ran for an additional 30 min on a treadmill with continuous monitoring of their heart rate, blood pressure, and O_2_ consumption. The experiments were carried out indoors at the Sports Diagnostics, Life Style, and Therapy Center of the University of Debrecen. Swimming was not included into the exercise session, as there is no appropriate swimming pool within the facility. The running platform had an inclination of 5 degrees to simulate the air resistance experienced during outdoor running. The subjects were asked to control their performance as they would do when doing the cycling and running disciplines of a triathlon event. Furthermore, during the entire exercise protocol, the coach of the triathlon team was standing next to the exercising athletes and supervised how they distribute their power. The team members were at the time of their training period, therefore the intensity of exercise load both on the bike and treadmill was set as to be around 60% of their maximal O_2_ consumption rate (measured before the 4-wk-long session) except for the last 5 min where they were asked to run as fast as they are able to simulate the finish of an actual contest. All exercise protocols were conducted at 9:00 AM and participants had their assigned breakfast at 7:00 AM. Similarly, blood samples at all recovery time points were again taken at 9:00 AM with the breakfast at 7:00 AM.

Due to the ethical regulations, experiments involving intensive exercise cannot be performed in Hungary without a prior cardiac ultrasound examination. Not only did we meet the requirements, but to be able to observe any possible alterations caused by the exercise load, echocardiography examinations were performed on all four athletes at three different time points. The baseline examination was recorded after the racing season, whereas the two others were performed in the preparation time: one immediately after endurance exercise and the last one was done 1 wk later. Descriptions of the echocardiography examination and three-dimensional (3-D) measurements are given in the following sections. The result of left and right ventricular analyses can be found in the Supplemental Table S1.

### Echocardiography

Three-dimensional echocardiography (3DE) was acquired using an Epiq 7C (Philips Medical Systems, Andover, MA) equipped with an X5-1 transducer. Patients were scanned in the left lateral decubitus position on an examination bed with a dedicated left-sided cutout, which allows optimal probe access for apical right ventricle (RV)-focused acquisitions. Full-volume acquisitions of the RV were performed from the RV-focused apical view during a single breath-hold using second-harmonic imaging, and the full-volume acquisitions of the left ventricle (LV) were performed from the apical view. The gain settings were optimized before the data acquisition. The temporal resolution was maximized by optimizing the sector width and minimizing the depth. The image optimization maneuvers were implemented as previously described (9). Whenever possible, imaging included 6-beat 3-D full-volume data sets focused on the desired chamber in one single breath hold. All 3-D data sets were digitally stored and analyzed offline. Three-dimensional data sets were digitally stored and analyzed offline.

### Measurements

3DE data sets of the RV and LV were analyzed offline to measure the end-diastolic and end-systolic volumes [indexed it on body surface area: end-diastolic volume index (EDVi), end-systolic volume index (ESVi)], and the ejection fraction (EF) of the right and left ventricle using a commercial software package (4D LV-Function 3, 4D RV-Function 2, TomTec Imaging Systems GmbH, Unterschleissheim, Germany). All of the volumetric analyses were performed by an experienced cardiologist.

### Blood Sampling

Blood samples were taken right before (Pre), right after the exercise (Post), and then 1 (Post1), 2 (Post2), and 7 days (Post7) later to follow restitution. Blood collection was performed by a professional healthcare nurse. Briefly, after skin disinfection, BD Vacutainer blood drawing system was used. Plastic EDTA tubes (BD Vacutainer, Cat. No.: 367863) were used for total blood count, plastic serum, and glass plasma tubes for additional examinations (BD Vacutainer, Cat. No.: 367815, 369714), and PAXgene Blood RNA tubes for whole blood RNA isolation (BD Biosciences, Cat. No.: 762165). The total volume was ∼15 mL for each blood drawing. The qualitative cell counts in the blood samples were determined using a Sysmex XN-2000 hematology analyzer (Sysmex Corporation, Kobe, Japan). Routine laboratory parameters in serum and urine samples were measured at the Department of Laboratory Medicine, University of Debrecen.

### RNA-Seq Method

To obtain transcriptome data of coding and different types of noncoding polyadenylated RNAs, high-throughput RNA sequencing analysis was performed on Illumina sequencing platform. Total RNA sample quality was checked on Agilent BioAnalyzer using Eukaryotic Total RNA Nano Kit according to the manufacturer’s protocol. Samples with RNA integrity number (RIN) value >7 were accepted for library preparation process. RNA-Seq libraries were prepared from total RNA using Ultra II RNA Sample Prep kit (New England BioLabs) according to the manufacturer’s protocol. Briefly, poly(A) RNAs were captured by oligo-dT conjugated magnetic beads then the RNAs were eluted and fragmented at 94°C. First-strand cDNA was generated by random priming reverse transcription and after second-strand synthesis step double-stranded cDNA was generated. After repairing ends, A-tailing and adapter ligation steps adapter-ligated fragments were amplified in enrichment PCR, and finally, sequencing libraries were generated. Sequencing run were executed on Illumina NextSeq 500 instrument using single-end 75 cycles sequencing.

### RNA-Seq Data Analysis and Identification of Differentially Expressed Genes

Raw sequencing data (fastq) was aligned to human reference genome version GRCh38 using HISAT2 algorithm and binary alignment and map (BAM) files were generated. Downstream analysis was performed using StrandNGS software (www.strand-ngs.com). BAM files were imported into the software, and DESeq algorithm was used for normalization. The normalized counts were log-transformed and base-lined to the data set resulting in “normalized signal values.” The significantly differentially expressed genes (DEGs) were identified by using one-way ANOVA with post hoc Tukey’s honestly significant difference (HSD) test in all possible pairwise comparisons with *P* < 0.05 significance level.

### Exploratory Data Analysis

Clustered heat map generation, principal component analysis, and Venn diagrams were generated by the iDEP v0.95 software, a Shiny app powered by R/Bioconductor packages (http://bioinformatics.sdstate.edu/idep95/). For heat map generation and hierarchical clustering analysis the expression data were normalized by EdgeR and the log2(CPM + c) transformed data were used. All transcripts were used in hierarchical clustering using the heatmap.2 function. The data were centered by subtracting the average expression level for each gene and the distance matrix was 1 − *r*, where *r* is Pearson’s correlation coefficient. The average linkage was used to generate the heat map and the corresponding hierarchical clustering tree.

### Gene Set Enrichment Analysis

The Web-based Gene Set AnaLysis Toolkit (Webgestalt; http://www.webgestalt.org) software was used to carry out gene set enrichment analysis (GSEA). The list of the differentially expressed genes was ranked by using the −log10 *P* value multiplied by the sign of log-transformed fold change obtained from the differential gene expression analysis as it was suggested by Reimand et al. (10). For pathway enrichment analysis the Reactome pathway database was used. The normalized enrichment score (NES) was calculated to characterize the enrichment of a pathway in our list of genes of interest, and the significance of NES was characterized with a permutation-based false discovery rate (FDR) value. In our study, the FDR ≤ 0.05 was used as the pathway significance threshold.

### Statistical Analysis

Statistical analyses were performed with SPSS 24.0 for Windows (Statistical Product and Service Solutions, v. 24, SPSS Inc., Chicago, IL). Normality was assessed with a normal probability (Q-Q) plot and with a Shapiro–Wilk test. All continuous variables are reported as the mean ± standard deviation (SD). The analyses for continuous data were performed with independent *t* test or one-way ANOVA followed by Bonferroni’s multiple comparison test. Non-normally distributed values are expressed as the median (interquartile range, IQR) and were compared using Mann–Whitney *U* test or Kruskal–Wallis test. A value of *P* < 0.05 was accepted as indicative of statistical significance, and all *P* values were two-sided.

## RESULTS

### Anthropometric and Cardiovascular Data

To exclude the possible effects of age and sex, only young female athletes were recruited for this study. Anthropometric and physiological parameters of athletes are presented in Table 1. The heart rates and blood pressure values before, during, and 5 min after the end of the exercise are displayed in Table 2 for each participant. These data demonstrate that the measured parameters of enrolled participants were quite similar.

**Table 1.** Anthropometric and physiological parameters

Parameter	Value
Age, yr	15.3 ± 1.3
Body weight, kg	51.5 ± 5.3
Height, cm	164.3 ± 8.0
Body mass index, kg/m^2^	19.1 ± 1.6
Normalized maximal O_2_ consumption rate, kg^−1^·mL^−1^·min^−1^	58.6 ± 3.6

Values are presented as means ± SD (*n* = 4).

**Table 2.** Cardiovascular parameters of the participants during the exercise session

Participant	Parameter	Before	During&	After$
1	Heart rate, beats/min	52	194	80
	Blood pressure, mmHg*	114/61	160/93	124/76
2	Heart rate, beats/min	60	200	83
	Blood pressure, mmHg*	109/63	158/69	114/78
3	Heart rate, beats/min	55	195	107
	Blood pressure, mmHg*	116/63	164/98	129/79
4	Heart rate, beats/min	63	201	99
	Blood pressure, mmHg*	125/62	138/90	129/78

* Systolic/diastolic values; &highest value measured during the exercise; $5 min after the end of the exercise.

### Blood Cell Counts and Biochemical Data

**Figure 1.**
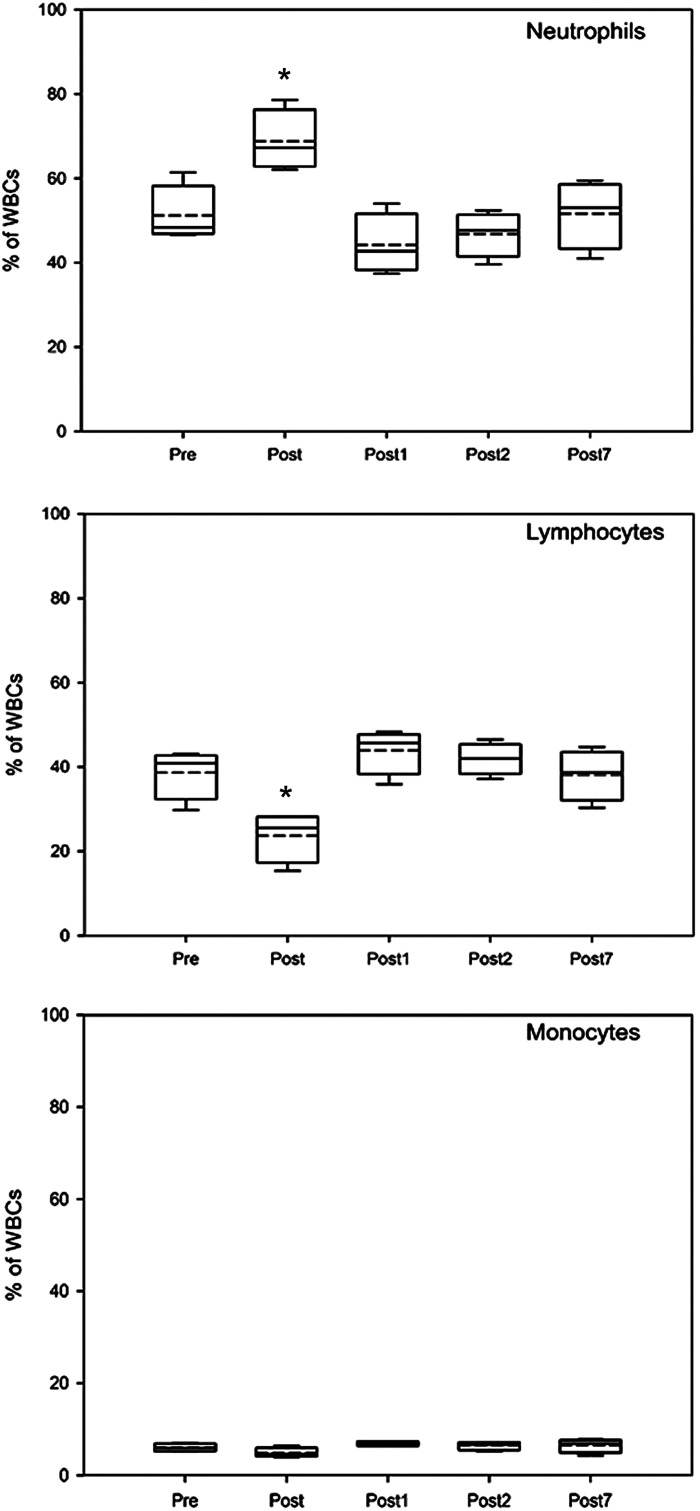
Effects of endurance exercise on the percentage of neutrophils, lymphocytes, and monocytes. Blood samples were taken right before (Pre), right after the exercise (Post), and then 1 (Post1), 2 (Post2), and 7 days (Post7) later. In the statistical analysis, one-way ANOVA followed by Bonferroni’s multiple comparison test was used with significance defined as *P < 0.05. WBCs, white blood cells (*n* = 4).

The exercise session led to a rapid increase in the total number of leukocytes (Table 3). The most remarkable changes were observed for neutrophils and lymphocytes. The number of neutrophils increased by 1.40 × 10^3^ cells/µL, whereas the number of lymphocytes decreased by 0.52 × 10^3^ cells/µL (Table 3). The percentage of neutrophils elevated from 51.2 ± 6.9% to 68.8 ± 7.2% (*P* = 0.011), whereas that of lymphocytes decreased from 38.7 ± 6.03% to 23.7 ± 6.06% (*P* = 0.012; Fig. 1). The endurance exercise had no significant effect on the tested serum and urine parameters (Supplemental Table S2).

**Table 3.** Effects of exercise on the number of white blood cells

Cell Type	Pre	Post	Post1	Post2	Post7
WBC	5.62 ± 0.93	7.54 ± 2.71	4.79 ± 1.01	4.98 ± 1.30	5.33 ± 1.38
Neutrophils	3.02 (2.42–3.13)	4.42 (3.64–7.91)***	2.34 (1.50–2.57)	2.40 (1.57–3.19)	2.94 (1.79–3.77)
Lymphocytes	2.19 ± 0.58	1.67 ± 0.21	2.09 ± 0.49	2.05 ± 0.37	1.97 ± 0.31
Monocytes	0.35 ± 0.11	0.37 ± 0.15	0.33 ± 0.06	0.32 ± 0.11	0.34 ± 0.11
Eosinophils	0.09 ± 0.03	0.06 ± 0.02	0.10 ± 0.06	0.08 ± 0.05	0.07 ± 0.03
Basophils	0.02 ± 0.02	0.03 ± 0.02	0.02 ± 0.01	0.02 ± 0.01	0.02 ± 0.01

Values are presented as means ± SD or median (interquartile range) (×10^3^ cells/µL). Blood samples were taken right before (Pre), right after the exercise (Post), and then 1 (Post1), 2 (Post2), and 7 days (Post7) later. In the statistical analysis, one-way ANOVA followed by Bonferroni’s multiple comparison test and Kruskal–Wallis test were used with significance defined as *P* < 0.05; ***P < 0.001 versus Pre (*n* = 4). WBC, white blood cells.

### Preliminary Transcriptome Analysis and Hierarchical Clustering

**Figure 2.**
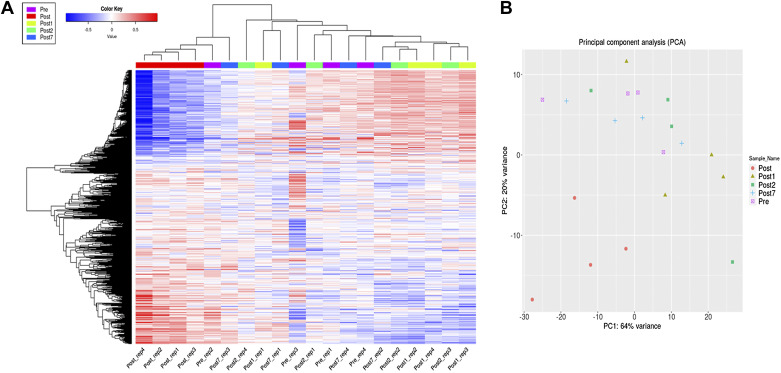
Heat map plot and hierarchical clustering for temporal profiling of the expression of the full data set of 2,795 transcripts present in all samples taken at different time points. *A*: hierarchical clustering dendrogram with heat map depicting the expression values of genes. The row dendrogram on the *left* of the heat map panel shows gene clusters, individual samples are denoted below the expression columns and labeled by a color code (*top* leftmost corner) in the column dendrogram, too. Blue indicates low expression, and red indicates high expression values. The time points of individual sample acquisition: Pre_rep1-4; Post_rep1-4; Post1_rep1-4; Post2_rep1-4; Post7_rep1-4: samples of the four participants taken right before (Pre), right after the exercise (Post), and then 1 (Post1), 2 (Post2), and 7 days (Post7) later, respectively. Expression data were generated by using total RNA sequencing analysis. *B*: principal component analysis applied to the complete data set replicates the separation of samples of the four individuals taken at Post time based on the gene expression data.

To assess the effect of endurance exercise on gene expression pattern, total RNA samples were isolated from peripheral blood samples of each participant at five different time points. RNA sequencing was used to identify the presence and determine the quantity of RNA transcripts in the samples. In our RNA-Seq data 10,891 transcripts were identified in the five separate sets of samples, out of them 2,795 showed significantly different expression in at least one pairwise comparison of the five different sample acquisition time points based on their ANOVA *P* < 0.05 values. Finally, 1,855 transcripts were annotated to unique Ensemble IDs, and this gene list was used for further analysis (Supplemental Table S3). To visualize the changes in gene expression pattern during the time course of the experiment, the transformed expression values of transcripts present in samples of the four athletes taken at the different time points were used to generate heat map with hierarchical clustering. Furthermore, a principal component analysis (PCA) was carried out to provide information about the overall distribution of gene expression values (Fig. 2).

The results of clustering methods and the PCA show that the endurance exercise induced specific and highly similar gene expression pattern in all four athletes. The samples taken right after the endurance exercise (Post_rep1-4 samples) are clustered together based on their expression pattern in the heat map and in the hierarchical clustering tree and separated from all other samples by the first two principal components in PCA. The rest of the samples show more or less random clustering and distribution in the PCA, suggesting that the gene expression values present in individuals before the exercise or following a resting period of different length do not show any specific pattern. This distribution might indicate that endurance exercise does not have long-lasting effect on gene expression, and differences seen in the heat map are very likely due to differences in basic gene expression profiles of the participants (Fig. 2).

### Differential Gene Expression Analysis

Among the identified 1,855 DEGs 949 genes showed significantly differential expression values in samples measured before and immediately after the endurance exercise (Post vs. Pre comparison), and 1,678 DEGs were identified in Post1 versus Post comparison (Table 4). Among the 1,855 DEGs 772 were common in both comparisons. Out of the 949 DEGs of the Post versus Pre comparison 670 were upregulated and 279 were downregulated. Among the 1,678 DEGs found in the Post1 versus Post comparison, 414 showed upregulation, whereas 1,264 showed downregulation (Table 4). When the expression values of these 1,678 genes were analyzed in the Post1 versus Pre comparison 439 transcripts were upregulated and 1,240 transcripts were downregulated. In all other pairwise comparisons, there were small, nonsignificant changes in the differential expression of genes. The similar distribution of up- and downregulated DEGs in the Post1 versus Post and Post1 versus Pre comparisons suggests that after a 24 h-long resting period, the expression of DEGs did not completely return to their original basic level. We have to note, however, that for most of the differentially expressed transcripts the changes in the expression were modest.

**Table 4.** The number and distribution of the significantly differentially expressed genes identified by pairwise comparison of samples taken at three different time points

Pairwise Comparison of Gene Expression	DE Genes	Upregulated Genes	Downregulated Genes
Post vs. Pre	949	670	279
Post1 vs. Post	1,678	414	1,264
Post1 vs. Pre	1,678	439	1,240

Samples were taken right before (Pre), right after the exercise (Post), and 1 day after the exercise (Post1).

### Functional Enrichment Analysis of DEGs by Gene Set Enrichment Analysis

**Figure 3.**
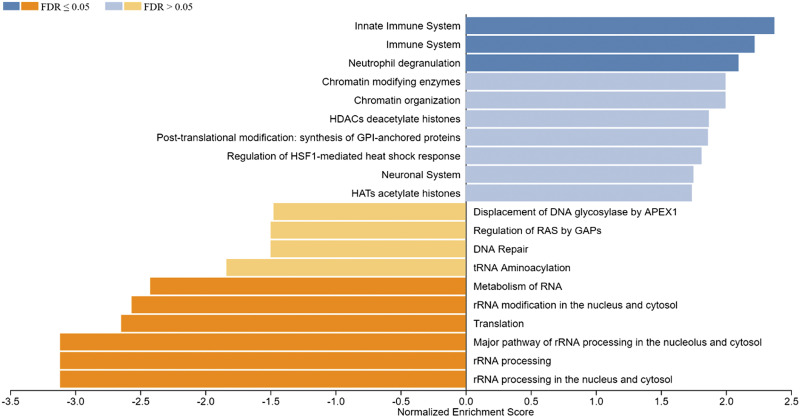
Gene set enrichment analysis (GSEA) of ranked significant differentially expressed genes of the Post vs. Pre comparison (949 genes) using the Reactome pathway database. Normalized enrichment scores indicate the distribution of the analyzed categories (*y*-axis) across our list of ranked DEGs. Higher positive (blue bars) or negative (yellow bars) enrichment scores (*x*-axis) represent up- or downregulation, respectively. The color code on the *top left* corner represents FDR values. DEGs, differentially expressed genes; FDR, false discovery rate; GAPs, GTPase-activating proteins; HDACs, histone deacetylases.

**Figure 4.**
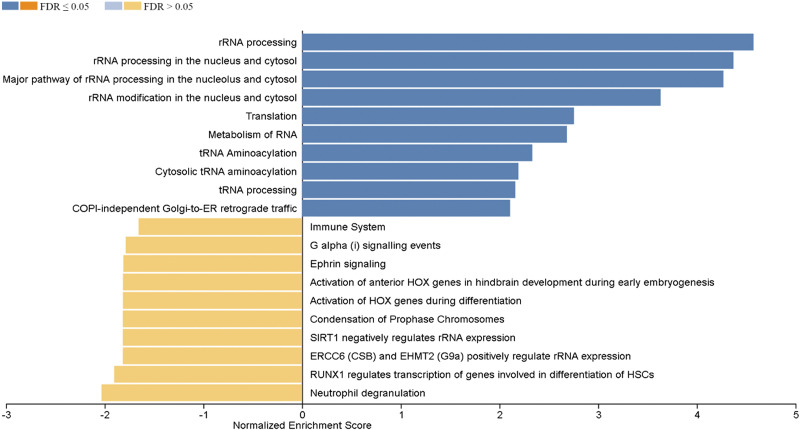
Gene set enrichment analysis (GSEA) of significant differentially expressed genes in Post1 vs. Post comparison (1,678 genes) using the Reactome database. Normalized enrichment scores indicate the distribution of the analyzed categories (*y*-axis) across our list of ranked DEGs, higher positive (blue bars) or negative (yellow bars) enrichment scores (*x*-axis) represent up- or downregulation, respectively. The color code on the *top left* corner represents FDR values. COPI, coat protein complex I; DEGs, differentially expressed genes; ER, endoplasmic reticulum; FDR, false discovery rate.

The WebGestalt software was used to carry out detailed functional enrichment analysis in our ranked list of DEGs identified in the Post versus Pre and Post1 versus Post comparisons using the Reactome database. Our ranked list of DEGs obtained for the Post versus Pre comparison contained 949 genes. We have applied the calculated normalized enrichment score (NES) value to characterize the enrichment of a pathway in our gene list, and the statistical significance of the enriched pathway was reflected by a permutation-based FDR value. The Reactome database GSEA revealed that the top three, significantly upregulated pathways enriched in our gene sets were the innate immune system, immune system, and neutrophil degranulation. Interestingly gene sets coding for chromatin modifying enzymes, histone deacetylase enzymes, and chromatin organization were also upregulated. These processes could lead to changes in epigenetic markers that are known to affect gene expression. Among the significantly downregulated enriched gene sets rRNA processing in the nucleus and cytosol, rRNA processing, major pathway of rRNA processing in the nucleolus and cytosol, translation, rRNA modification in the nucleus and cytosol, and metabolism of RNA can be found (Fig. 3). We have also carried out GSEA using the ranked list of DEGs obtained in the Post1 versus Post comparison using again the Reactome pathway database. The top 10 enriched Reactome pathways obtained for DEGs in this comparison are displayed in Fig. 4. The results of the GSEA analysis showed that the top five enriched upregulated Reactome pathways were rRNA processing, rRNA processing in the nucleus and cytosol, major pathway of rRNA processing in the nucleolus and cytosol, rRNA modification in the nucleus and cytosol, and translation. The top five downregulated pathways were neutrophil degranulation, RUNX1 regulates transcription of genes involved in differentiation of HSCs, ERCC6 (CSB), and EHMT2 (G9a) positively regulate rRNA expression, SIRT1 negatively regulates rRNA expression and condensation of prophase chromosomes. It is easy to notice that pathways that were upregulated in the Post versus Pre comparison were downregulated in the Post1 versus Post comparison, suggesting that the endurance exercise and the 24-h long resting period oppositely affected the gene expression pattern. The endurance exercise typically upregulated the expression of genes acting in immune processes, whereas downregulated genes of rRNA metabolism, ribosome biogenesis, and RNA metabolism, suggesting that protein synthesis is reduced as a result of endurance exercise. However, the 24-h long resting period quickly reverted this gene expression pattern.

**Figure 5.**
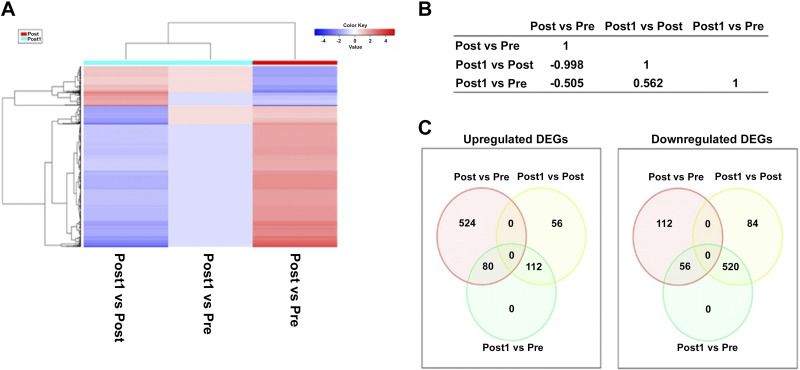
Heat map with hierarchical clustering dendrogram of the expression values of the 772 differentially expressed genes common in three pair-wise comparisons: Post vs. Pre: change between expression values measured right after endurance exercise and before endurance exercise; Post1 vs. Post: change between expression values measured at 24 h after endurance exercise and right after endurance exercise; Post1 vs. Pre: change between expression values measured at 24 h after endurance exercise and right before endurance exercise. *A*: hierarchical clustering with heat map dendrogram. Gene clusters are shown on the *left*, and individual samples are denoted below the expression columns. Each sample listed contained the average gene expression value of the four participants. Upregulated genes are red, downregulated genes are blue. *B*: pairwise correlation matrix data between the average gene expression values in the three data sets. Pearson’s correlation coefficient values are shown in the table. *C*: Venn diagrams showing the overlap between the upregulated and downregulated significantly differentially expressed genes in Post vs. Pre, Post1 vs. Post, and Post1 vs. Pre pairwise comparisons.

**Figure 6.**
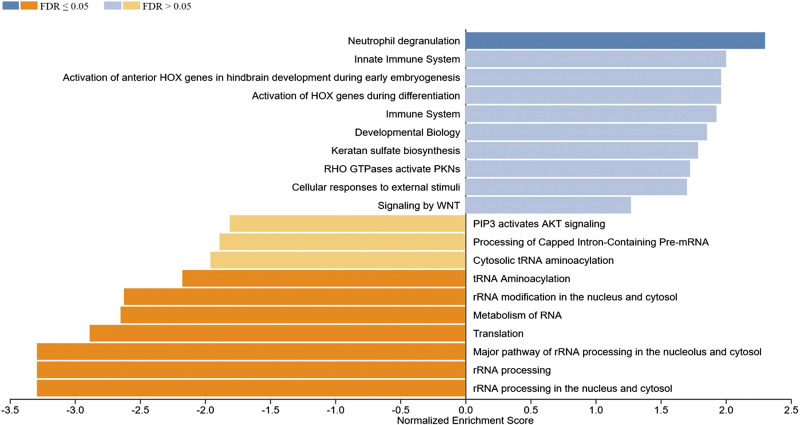
Gene set enrichment analysis of differentially expressed 772 genes in Post vs. Pre comparison. Bar plot of pathway analysis using the Reactome pathway database. Normalized enrichment scores indicate the distribution of the analyzed categories (*y*-axis) across our list of ranked DEGs, higher positive (blue bars) or negative (yellow bars) enrichment scores (*x*-axis) represent up- or downregulation, respectively. The color code on the *top left* corner represents FDR values. DEGs, differentially expressed genes; FDR, false discovery rate; PKNs, protein kinases N.

**Figure 7.**
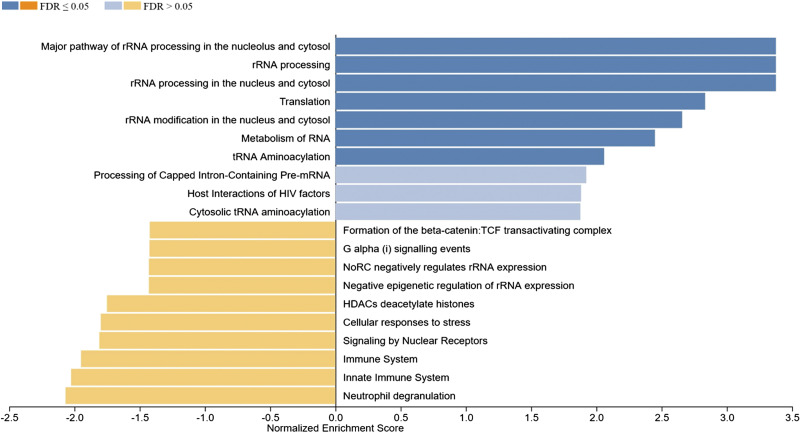
Gene set enrichment analysis of differentially expressed 772 genes in Post1 vs. Post comparison. Bar plot of pathway analysis using the Reactome pathway database. Normalized enrichment scores indicate the distribution of the analyzed categories (*y*-axis) across our list of ranked DEGs, higher positive (blue bars) or negative (yellow bars) enrichment scores (*x*-axis) represent up- or downregulation, respectively. The color code on the *top left* corner represents FDR values. DEGs, differentially expressed genes; FDR, false discovery rate; HIV, human immunodeficiency virus; NoRC, nucleolar remodeling complex; TCF, T-cell factor.

To investigate this contrasting gene expression pattern, a heat map dendrogram was created using average gene expression values of the 772 DEGs found to be common in the Post versus Pre, Post1 versus Post, and Post1 versus Pre comparisons for all participants. Venn diagrams were also produced to visualize the overlap of differentially expressed genes in given comparisons (Fig. 5). Out of the 772 genes there were 604 upregulated and 168 downregulated DEGs in the Post versus Pre comparison, whereas these genes have exactly the opposite expression pattern in the Post1 versus Post comparison suggesting that the observed response to endurance exercise on gene expression level is reverted during the resting period (Fig. 5*C*). GSEA using the 772 DEGs in the Post versus Pre comparison identified several gene sets enriched in our list of DEGs by Reactome pathway analysis. The top 10 up and downregulated sets for both databases are summarized in Fig. 6. The results of this analysis are similar to that of the previous GSEA analysis. According to our results, the Reactome pathway analysis revealed that the gene set of neutrophil degranulation was significantly upregulated in our list of genes. The FDR values of the rest of the upregulated sets remained below the significance level, among these gene sets the innate immune system, activation of anterior *HOX* genes in hindbrain development during early embryogenesis, activation of *HOX* genes during differentiation, immune system were found. However, several strongly and significantly downregulated gene sets were identified. The most significant sets are rRNA processing in the nucleus and cytosol, rRNA processing, major pathway of rRNA processing in the nucleolus and cytosol, translation, and metabolism of RNA. The physiological function of these sets suggests that genes downregulated by endurance exercise were primarily enriched in pathways of RNA processing, and metabolism of rRNA and tRNA, suggesting that translation is strongly affected by endurance exercise. Immune functions and, interestingly, gene sets that are involved in the differentiation of certain cell types were upregulated. The results of the GSEA of the Post1 versus Post comparison of the above-analyzed 772 DEGs using the Reactome pathway database are shown in Fig. 7. This analysis provided a more or less reversed sequence of the up and downregulated gene sets compared with the Post versus Pre comparison. This confirms our previous notion, that a 24-h long resting period reverses the gene activation pattern generated by endurance exercise.

### Analysis of Immune Cell Signature

**Figure 8.**
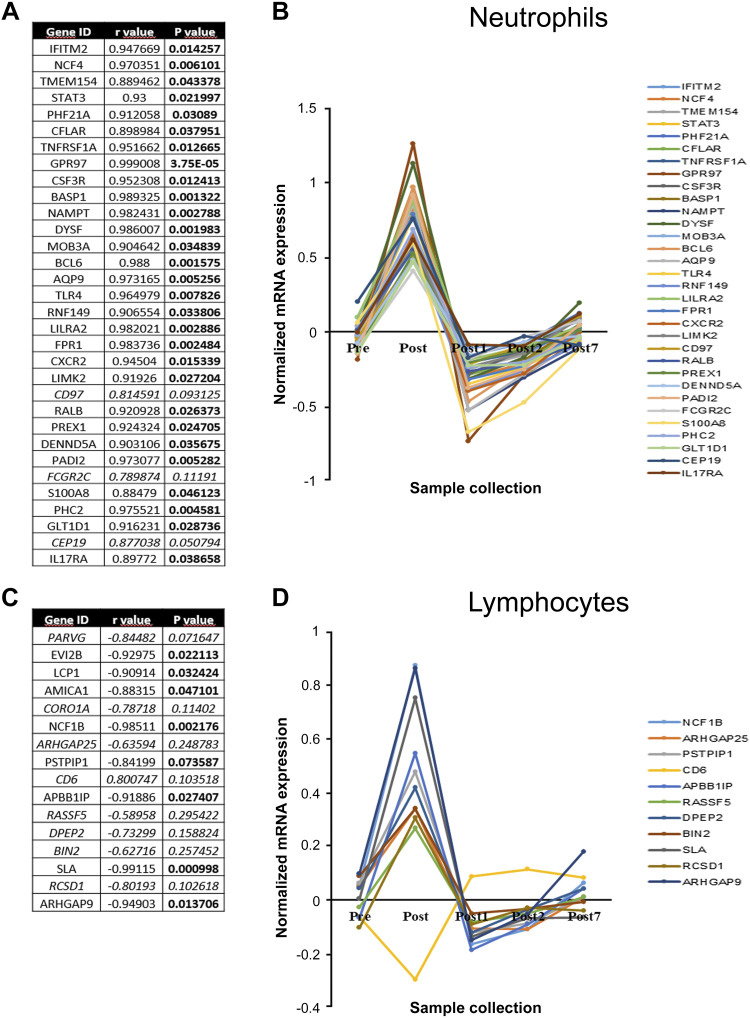
Analysis of immune cell signature. *A* and *C*: list displayed of overlapping genes between ImSig and our transcriptomic data. The correlation between changes in the ratio of the given cell type among all white blood cells and gene expression values is expressed as the Pearson correlation coefficient (*r* value). *P* values represent the significance of the correlation. *B* and *D*: kinetics of changes in gene expression values normalized to the alterations in cell percentage.

To investigate immune cell type-specific gene expression changes associated with the response to endurance exercise, the ImSig set of immune cell gene signatures was applied (11). The analysis focused on neutrophils and leukocytes, the percentage of which significantly changed after exercise session. First, the number of overlapping genes between ImSig and our transcriptomic data was determined. It was found that 32 out of 42 neutrophil marker genes and 16 out of 85 T-cell marker genes showed significant differential expression right after the exercise (Fig. 8). Next, the relationship between gene expression changes and kinetics of cell ratio alterations was examined. In the case of neutrophils, changes in the expression of overlapping genes were strongly and positively correlated with the kinetics of alterations in neutrophil cell percentage (Fig. 8). However, there were three genes (*BCL6*, *DYSF*, and *GPR97*) whose expression enhanced more after exercise than the proportion of neutrophil cells increased. In the case of T cells, the detected changes in the expression of overlapping genes were negatively correlated with the kinetics of alterations in lymphocyte percentage (Fig. 8). The only exception to this phenomenon was *CD6*, the expression of which was in line with changes in lymphocyte percentage (Fig. 8). For this reason, *CD6* was excluded from further analysis.

**Figure 9.**
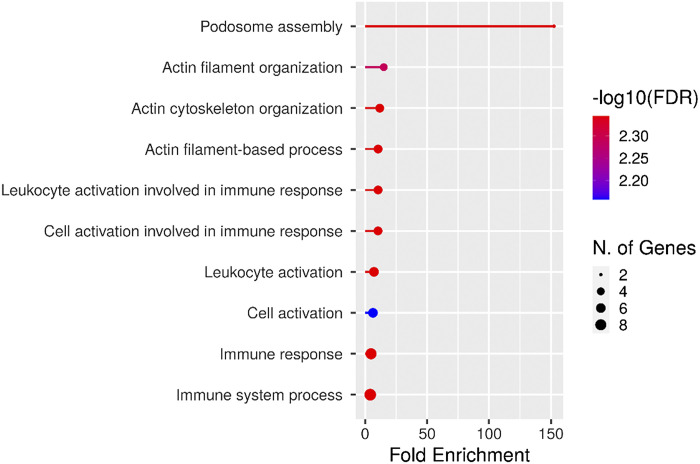
Top 10 significant overrepresented pathways revealed after functional enrichment analysis of the overlapping genes between ImSig lymphocyte gene signature and our transcriptomic data.

To determine the Gene Ontology (GO) terms of biological functions, which are significantly overrepresented in the overlapping genes (*PARVG, EVI2B, LCP1, AMICA1, CORO1A, NCF1B, ARHGAP25, PSTPIP1, APBB1IP, RASSF5, DPEP2, BIN2, SLA, RCSD1*, and *ARHGAP9*), ShinyGO (v0.75) online tool was used. The top 10 biological process GO terms are visualized in Fig. 9. Among these GO terms podosome assembly, actin filament organization, actin cytoskeleton organization, and actin filament-based process were the most represented. These findings suggest that the overlapping genes listed earlier play a role in the active alteration of cell shape and, consequently, the adhesion of lymphocytes to the vessel wall.

## DISCUSSION

In this study, cardiovascular, hematological, and biochemical parameters, as well as the results of whole blood mRNA sequencing have been evaluated to reveal the responses of young female athletes to an exercise session mimicking the cycling and running disciplines of a junior triathlon competition. It has been found that the applied exercise load has not caused remarkable changes in cardiovascular or biochemical parameters of trained, young elite athletes; however, induced a significant increase in the percentage of neutrophils and a significant drop in the ratio of lymphocytes in the blood immediately after endurance exercise.

Previous studies have also identified rapid elevations in blood neutrophil counts and ratios after acute exercise (12–14). In general, the exercise-induced neutrophilia is thought to be a result of increased shear stress associated with enhanced blood flow and that of demargination of cells from endothelium initiated by stress hormones such as catecholamines (15). These events are followed by a cortisol-induced mobilization of immature neutrophils from the bone marrow (16). Moreover, neutrophils can also be mobilized in response to the secretion of proinflammatory cytokines (mainly IL-6) triggered by exercise-induced muscle damage (17).

During vigorous exercise sessions, it is also commonly observed that peripheral blood lymphocyte frequency is dramatically increased due to hemodynamic shear stress and/or catecholamines acting on β_2_-adrenergic receptors (18). However, following prolonged and/or high-intensity exercise in particular, lymphocyte number decreases to below the pre-exercise level in less than 30 min (18). In our experiments, a significant decrease in the percentage of lymphocytes was detected immediately after the endurance exercise. This phenomenon may be due to the fact that the young athletes performed the last 5 min of the exercise session at maximum intensity. This assumption is supported by previous findings that a drop in lymphocyte count could be observed right after exhaustive exercise (19). A growing body of evidence suggests that this acute and transient lymphopenia after exercise is beneficial to immune surveillance and regulation (20). Indeed, it is widely proposed that acute exercise redeploys lymphocytes to peripheral sites of potential antigen encounter (e.g., mucosal surfaces). There, these immune cells are thought to identify and eradicate pathogens as well as infected and/or damaged cells. It is hypothesized that this redistribution reflects heightened immune surveillance in tissues and organs where pathogens are likely to be encountered during and after exercise (i.e., lungs, gut; 21).

The effects of acute and long-term exercise on peripheral blood gene expression have been reported in a number of studies, of which in more than 20 genome-wide mRNA profiling methods have been used (reviewed in Ref. 8). In the vast majority of these studies, leukocytes or leukocyte subsets (peripheral blood mononuclear cells/PBMCs/, lymphocytes, monocytes, and neutrophils) were investigated and genome-wide gene expression data sets were generated using microarray technology. In this study, RNA-seq to whole blood samples was utilized to explore kinetic profile of the transcriptome changes induced by endurance exercise. A significant number (nearly 950) of genes that are up- or downregulated as a result of our exercise protocol were identified. Pathway enrichment analysis revealed that the significantly upregulated pathways enriched in our gene sets were the innate immune system, immune system, and neutrophil degranulation, whereas the significantly downregulated ones were rRNA processing in the nucleus and cytosol, rRNA processing, major pathway of rRNA processing in the nucleolus and cytosol, translation, rRNA modification in the nucleus and cytosol, and metabolism of RNA. These observations are consistent with the results of a recent study. Glotov et al. (22) applied whole blood RNA-seq to seven elite female skaters, to assess the immunological and metabolic effects of high-altitude adaptation. They found that inflammatory and immune pathways dominated the observed changes, and on a cellular level, neutrophil degranulation accounts for a substantial fraction of upregulated genes. The majority of pathways enriched among the downregulated genes were related to ribosomal proteins and other components of transcriptional and translational machinery (22). Despite different exercise loads applied, the consistency of our results with the gene expression data of this another study (22) suggests that whole blood RNA-seq is a useful tool for monitoring physiological effects of various exercise loads.

The ImSig immune cell signature has been developed to facilitate the characterization of tumor microenvironment’s immune composition (11). The differentiation or activation state of immune cells within tissues and in the blood may be different, and consequently markers derived from tumor tissues do not necessarily transfer well to blood cells. However, a number of previous findings suggest that endurance exercise affects not only leukocyte counts but also responsiveness, indicating an altered activation state of the circulating cells (23). Moreover, a recent study has found that an individual’s whole blood transcriptome can significantly predict tissue-specific expression levels for ∼60% of the genes on average across 32 tissues, suggesting a close association between tissue-specific gene expression and whole blood transcriptomics (24). Indeed, applying ImSig it was revealed that 32 out of 42 neutrophil marker genes showed significant differential expression immediately after the exercise session. However, for most of the 32 genes, an increase in the expression was detectable due to elevated neutrophil count. Only three genes were the exception (*BCL6, DYSF*, and *GPR97*), as their expressions were enhanced to a greater extent than the proportion of neutrophil cells increased. Proteins encoded by these genes play an important role in neutrophil survival and activation. It has been recently reported that BCL6 promotes survival of neutrophils by binding to the gene loci involved in apoptosis (25). It is important to note that an enhanced cell death following BCL6 deficiency is restricted to the activated neutrophils and does not occur in resting neutrophils in the blood or bone marrow (25). In activated neutrophils the specific and azurophil granules are mobilized, a process of importance for phagocytosis and intracellular killing of microbes by fusing primarily with the phagosome (26). Detergent-resistant membrane domains are present in the membranes of azurophil granules in human neutrophils. Dysferlin (DYSF) isolated from these lipid rafts is considered a protein with possible function in membrane fusion and maintenance (27). Dysferlin belongs to the ferlin family of proteins and contains C2 domains indicating its involvement in Ca^2+^-dependent membrane fusion events (27). The adhesion family of G protein-coupled receptors (aGPCRs) comprises 33 members in human, one of which is GPR97 (28). A recent work has indicated that GPR97/ADGRG3 is abundantly expressed in granulocyte precursors and terminally differentiated neutrophilic, eosinophilic, and basophilic granulocytes (29). GPR97 was also detected in tissue-infiltrating neutrophils and shown to be upregulated during systemic inflammation. Ligation of GPR97 increases reactive oxygen species production and proteolytic enzyme activity, leading to an increased killing activity of neutrophils (29).

Comparison of ImSig and our transcriptomic data identified 15 overlapping genes that are related to T-cell functions and may have a role in podosome formation and adhesion to the vessel wall. This observation is consistent with previous findings showing that during spreading and lateral migration, lymphocytes generate discrete actin-rich micron-scale projections that extend in the direction orthogonal to the plane of migration (30). These cylindrically shaped podosome-like structures protrude from the bottom of the T lymphocytes, exerting mechanical forces against the surface of the endothelium. Lymphocytes extend podosomes to palpate endothelial cells searching for areas permissive for transcellular diapedesis. Podosomes contain adhesion molecules, adaptors, and kinases resembling those found in focal adhesions linked to stress fibers. However, in contrast to focal adhesions, podosome assembly does not require de novo protein synthesis, and podosome cores are highly enriched in proteins involved in actin polymerization (31, 32).

Taken together, investigation of whole blood transcriptome identified highly similar gene expression patterns in the samples of all four young athletes after endurance exercise. The most significant differences in gene expression were observed within 24 h after the exercise session. Pathway enrichment analysis revealed that as a result of intense exercise, the expression of genes linked to the innate immune responses and the activation of neutrophil cells increased to the greatest extent. Whereas, intensive training primarily decreased the expression of genes associated with rRNA processing and translation. A 24-h long resting period almost completely reversed the gene activation pattern generated by exercise load. These observations together with the results of a recent study (22) suggest that whole blood RNA-seq is a useful tool for monitoring physiological effects of various exercise programs, albeit further studies are needed to confirm this assertion. Application of ImSig set of immune cell gene signatures helps to investigate immune cell type-specific gene expression changes associated with the response to endurance exercise. However, ImSig analysis can only be used for genes whose expression changes in the opposite way, or changes more than the change in the proportion of cells expressing them in the blood. Our results may contribute to a better understanding of the adaptation mechanisms of the young female body following intense physical exertion.

## DATA AVAILABILITY

The RNA-Seq data that support this study are available as follows: NCBI Sequence Read Archive (SRA), BioProject ID: PRJNA843596 (http://www.ncbi.nlm.nih.gov/bioproject/843596).

## SUPPLEMENTAL DATA

10.6084/m9.figshare.21435357 Supplemental Tables S1–S3: https://doi.org/10.6084/m9.figshare.21435357.

## GRANTS

This work was supported by the Thematic Excellence Programme (TKP2020-NKA-04) of the Ministry for Innovation and Technology in Hungary, by the GINOP-2.3.2-15-2016-00062 project cofinanced by the European Union and the Regional Development Fund, and by the National Research, Development and Innovation Office (K 131844 to S.B.).

## DISCLOSURES

No conflicts of interest, financial or otherwise, are declared by the authors.

## AUTHOR CONTRIBUTIONS

A.B., I.P., J.B., L.C., and I.B. conceived and designed research; G.B., S.B., E.G.K., and C.J. performed experiments; A.B., A.P., G.B., S.B., E.G.K., C.J., I.P., J.B., L.C., and I.B. analyzed data; A.B., A.P., S.B., C.J., I.P., J.B., L.C., and I.B. interpreted results of experiments; A.B., A.P., and S.B. prepared figures; A.B. and A.P. drafted manuscript; A.B., A.P., I.P., L.C., and I.B. edited and revised manuscript; A.B., A.P., G.B., S.B., E.G.K., C.J., I.P., J.B., L.C., and I.B. approved final version of manuscript.
